# Long-Term Immune Consequences of Initial SARS-CoV-2 A.23.1 Exposure: A Longitudinal Study of Antibody Responses and Cross-Neutralization in a Ugandan Cohort

**DOI:** 10.3390/vaccines13020143

**Published:** 2025-01-29

**Authors:** Gerald Kevin Oluka, Jackson Sembera, Joseph Ssebwana Katende, Violet Ankunda, Laban Kato, Ashwini Kurshan, Carl Graham, Jeffrey Seow, Katie J. Doores, Michael H. Malim, Julie M. Fox, Pontiano Kaleebu, Jennifer Serwanga

**Affiliations:** 1 Viral Pathogens Research Theme, Medical Research Council, Uganda Virus Research Institute and London School of Hygiene & Tropical Medicine (MRC/UVRI & LSHTM) Research Unit, Entebbe 26, Uganda; 2 Department of Immunology, Uganda Virus Research Institute, Entebbe 26, Uganda; 3 Department of Infectious Diseases, School of Immunology & Microbial Sciences, King’s College London, London SE1 9RT, UK

**Keywords:** immune imprinting, SARS-CoV-2 A.23.1 variant, cross-neutralization, antibody dynamics, neutralizing titers, antigenic surveillance, immune escape, variant-specific IgG immune responses

## Abstract

**Background:** This study assessed the long-term dynamics of neutralizing antibodies in a Ugandan cohort primarily exposed to the A.23.1 SARS-CoV-2 variant, examining how this shaped immune breadth and potency against diverse strains following infection and prototype-based vaccination. **Methods:** We conducted a 427-day retrospective analysis of 41 participants across multiple SARS-CoV-2 waves, assessing binding and neutralizing antibody responses using in-house ELISA and pseudotyped virus neutralization assays. We quantified immune responses to key SARS-CoV-2 variants, A.23.1, D614G, Delta, and BA.4, capturing evolving immunity across the pandemic. **Results:** Neutralizing antibody titers against A.23.1 remained significantly higher than those against D614G, Delta, and BA.4, highlighting the solid immune memory following A.23.1 infection. Consistently lower titers were observed for BA.4 across all time points, aligning with its strong immune-evasion capability. Correlations between neutralizing titers and spike-directed IgG (S-IgG) concentrations were significantly stronger for A.23.1 than for D614G, with no correlation for BA.4. ChAdOx1-S vaccination substantially elevated the neutralizing titers across all variants, most notably BA.4, highlighting the essential role of vaccination in boosting immunity, even in individuals with initially low titers. **Conclusions:** Initial exposure to the A.23.1 variant triggered potent immune responses, shaping neutralizing antibody dynamics during subsequent exposures. These findings highlight the importance of accounting for early viral exposures in vaccine development and public health planning. The distinctly lower immune response to BA.4 highlights the need for continuous antigenic monitoring and timely vaccine updates for protection against emerging variants. Vaccination remains essential for reinforcing and sustaining immunity against evolving variants.

## 1. Introduction

Immune imprinting, first described by Thomas Francis, Jr. in the context of influenza over fifty years ago, refers to how the immune system’s initial encounter with an antigen shapes future responses to future encounters with related antigens [1,2]. This concept has gained renewed interest in the study of emerging viral pathogens, including SARS-CoV-2 [3,4,5,6]. Research on variant-specific vaccines and successive epidemic waves has underscored the influence of initial immune priming on subsequent immune responses [5]. Understanding this phenomenon is essential for optimizing vaccine strategies and anticipating immune dynamics in response to evolving viral threats. Imprinting has been shown to have both beneficial [7,8,9] and detrimental effects [8,10]. Recent studies on SARS-CoV-2 variants show that initial immunization with multiple doses of the prototype mRNA-1273 vaccine effectively primes the immune system, enhancing broad cross-neutralizing antibody responses to subsequent Omicron-based boosters [11,12]. These findings underscore the critical role of early antigen exposure in shaping durable, broad-spectrum immunity against SARS-CoV-2.

**Figure 1:**
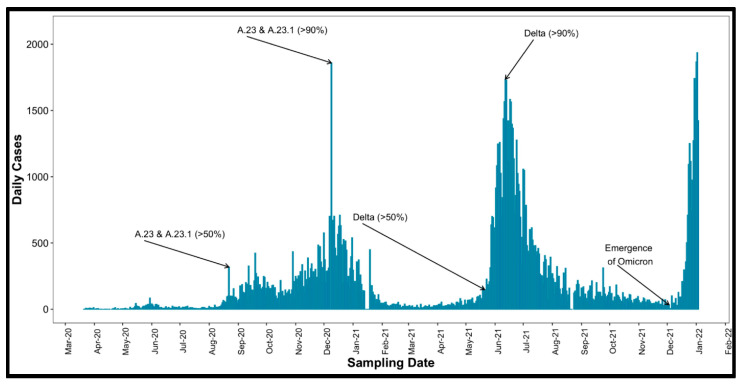
COVID-19 variant distribution and participant baseline characteristics. This figure shows the timeline of daily COVID-19 case counts and variant distribution from March 2020 to February 2022, indicating that samples collected between July 2020 and October 2021 are primarily linked to the A.23.1 and Delta variants. This figure is adapted and reused with permission from Bbosa et al. [21].

Uganda confirmed its first COVID-19 case on 21 March 2020 [13] and launched its vaccination campaign on 10 March 2021 after receiving 864,000 doses of the AstraZeneca vaccine. In August 2020, initial SARS-CoV-2 genome sequences from infection clusters in Uganda were identified as lineage A.23, which is characterized by spike protein mutations R1021, F157L, V367F, Q613H, and P681R. These constituted 32% of viruses sequenced from June to August 2020, increasing to 50% from September to November 2020. By late October 2020, the A.23.1 variant with an additional spike mutation (P681R) emerged [14], and from December 2020 to January 2021, 90% of identified genomes (102 of 113) belonged to the A.23.1 lineage [15,16]. Uganda’s Delta wave surged rapidly, rising from a daily average of 100 cases per day on 18 May 2021 to its peak at about 1800 cases per day by 12 June 2021, less than a month later. Between June and August 2021, the country recorded 2328 COVID-19 deaths, representing over half of its total mortality at the time. The Omicron wave, from December 2021 to January 2022, progressed even faster, peaking within just two weeks of onset at over 1800 cases per day [17], as summarized in Figure 1.

Our study uniquely investigated the A.23.1 variant, which constituted the primary antigenic exposure during Uganda’s initial SARS-CoV-2 outbreak [15]. A.23.1 is slightly distinct from the Wuhan-1 strain used in the vaccines administered in this population, owing to the presence of both V367F and Q613H mutations that increase its infectivity over the Wuhan-1 strain [18]. This, combined with the NTD mutations, F157L and R102I, likely created a unique immunological imprint on the Ugandan population, with the long-term effects on subsequent immune responses to natural infection and vaccination remaining largely unexplored. We addressed this gap by analyzing immune responses in a Ugandan cohort initially exposed to the A.23.1 variant, determining antibody binding in response to SARS-CoV-2 natural infection [19] and vaccines [20] using Wuhan-1 strain antigens. The impact of A.23.1 on subsequent infections and vaccine responses remains uncertain, as does the specificity of serum-binding antibody titers in targeting A.23.1. It is also unclear how these antibody levels correlate with neutralization of A.23.1 and other SARS-CoV-2 variants. Clarifying these interactions is essential for understanding the broader immune landscape shaped by natural infection and vaccination.

This study investigated how primary infection with the A.23.1 SARS-CoV-2 variant shaped antibody responses to both subsequent natural infections and vaccinations with the ancestral strain. By longitudinally tracking immune responses, we aimed to provide critical insights into the dynamics of antibody evolution in this context. Specifically, we assessed (1) how primary infection with the A.23.1 SARS-CoV-2 variant influenced the magnitude and breadth of serum-neutralizing antibody responses, (2) the correlation between binding antibody levels and neutralizing capacity against A.23.1 and other SARS-CoV-2 variants, and (3) the implications for immune assessments in this cohort. This is the first analysis of immune responses to a non-Wuhan-1 primary SARS-CoV-2 variant infection in an African population, and it potentially has implications for tailored vaccine development.

## 2. Materials and Methods

### 2.1. Study Design and Population

This retrospective study, based on a well-characterized COVID-19 cohort [19], included 41 participants monitored through subsequent variant epidemic waves (Figure 1). SARS-CoV-2-positive participants were identified through rt-PCR testing as part of Uganda’s national border surveillance, focusing on truck drivers who were essential to maintaining economic activity during the nationwide lockdown. This cohort provided a unique opportunity to study viral transmission in a high-risk, mobile population. During that period, all PCR-positive cases were mandatorily isolated at Masaka and Entebbe Regional Referral Hospitals until a confirmed PCR-negative result. This strict isolation policy was implemented to prevent further transmission. Infection onset (Day 0) was estimated using the initial rt-PCR confirmation documented in hospital records, as all PCR-positive truck drivers were mandatorily hospitalized. During follow-up, eight participants were vaccinated with the AstraZeneca ChAdOx1-S COVID-19 vaccine. Aligned with the emergence of COVID-19 variants and daily case counts (Figure 1), most samples collected between July 2020 and October 2021 primarily reflect immune responses to infections driven by the A.23.1 and Delta variants, which predominated during that period.

Of the 41 participants (median age: 29 years, IQR: 23–34), 78% were male. A majority (61%) were asymptomatic, 9.8% were symptomatic, and 29.3% had no symptom data (Table 1). Over a 427-day follow-up period (median 182.2 days, IQR 91.5–277.2), 156 plasma samples were collected at four intervals, which were 0–9, 92–182, 183–273, and 274–427 days post-PCR confirmation of infection. The study was approved by the Research and Ethics Committee of the Uganda Virus Research Institute (GC/127/833) and the Uganda National Council for Science and Technology (HS637ES). All participants provided written informed consent before their involvement.

### 2.2. Conventional In-House ELISA to Quantify Binding Antibody Concentrations

Anti-Spike-IgG antibodies were quantified using an optimized in-house ELISA protocol [22]. Briefly, 96-well flat-bottomed medium-binding plates (Greiner Bio-One, #655001, kremsmünster, Austria) were coated with 50 μL of recombinant wild-type spike-protein or nucleoprotein antigen (R&D Systems, #10474-CV-01M, #10474-CV-01M, Minneapolis, MN, USA) at 3.0 µg/mL and incubated overnight at 4 °C. The following day, plates were washed with PBS containing 0.05% Tween 20 (PBS-T) and blocked for 1 h at room temperature using PBS-T containing 1% BSA (Sigma, #A3803, Marlborough, MA, USA). Heat-inactivated plasma samples (56 °C for 30 min) were diluted 1:100 in PBS-T with 1% BSA and added in duplicate to the wells for 2 h of incubation at room temperature. After incubation, plates were washed five times with PBS-T, followed by one hour of incubation with 1:10,000 diluted horseradish peroxidase–conjugated goat anti-human IgG (γ-chain specific, Sigma, #A0170) in PBS-T with 1% BSA, at room temperature.

The assay included pre-determined negative and positive plasma samples, along with monoclonal antibodies CR3022 (0.1 µg/mL) targeting the spike protein and CR3009 (2 µg/mL) targeting nucleocapsid. Blank wells in duplicate served as controls. After washing, 50 μL of TMB substrate (Sera Care, #5120-0075, Milford, MA, USA) was added to each well and incubated for 3 min. The reaction was stopped by adding 50 μL of 1M hydrochloric acid (Sera Care, #5150-0021). Optical densities were measured at 450 nm, with blank well values subtracted to obtain net responses. Antibody concentrations were calculated using 4-parameter logistic standard curves and expressed in ng/mL, with values below the detection limit assigned 0 ng/mL. Longitudinal N-IgG concentrations were tracked to detect reinfection, defined by an 11-fold rise following the initial antibody peak, as previously reported [19].

### 2.3. Preparation of SARS-CoV-2 Pseudotyped Viruses and Assessment of Neutralizing Titers

Pseudotyped HIV-based viruses expressing SARS-CoV-2 spike variants D614G, B.1.617.2 (Delta), A.23.1, or Omicron (BA.4/5) were generated by culturing 3.5 × 10^6^ HEK293T/17 cells in 10 mL of complete Dulbecco’s Modified Eagle’s Medium (supplemented with 10% FBS and 1% Pen/Strep). The cells were co-transfected with 15 μg of pHIV-Luciferase plasmid, 10 μg of HIV-Gag/Pol p8.91 plasmid, and 5 μg of SARS-CoV-2 variant spike protein plasmid using 90 μg of Polyethylenimine (PEI-Max, Polysciences, PA, USA ) transfection reagent. After 18 h, the media were replaced, and the supernatant was collected 48 h post-transfection, filtered (0.45 μm), and stored at −80 °C. Heat-inactivated plasma samples were serially diluted five-fold starting at 1:25, in duplicates, using DMEM media with 10% FBS and 1% Pen/Strep. These were incubated with pseudotyped virus for 1 h at 37 °C in 96-well culture plates, followed by the addition of 12,500 HeLa cells stably expressing the human ACE-2 in 50 μL per well. The plates were incubated for 72 h at 37 °C with 5% CO_2_ in a humidified incubator. Infection inhibition was assessed using Bright-Glo luciferase (Promega, Madison, WI, USA) and quantified on a Victor™ X3 multilabel reader (Perkin Elmer, Waltham, MA, USA). The ID50 values, representing the dilution required to achieve 50% neutralization, were calculated using curve fitting with the dose–response drc package in R software, v4.4.1.

### 2.4. Statistical Methods

Boxplots displayed the medians, means, and quartiles, while the Wilcoxon test, adjusted with Bonferroni correction for multiple comparisons, assessed significant differences in neutralization titers. Spearman rank correlation evaluated the relationship between neutralization titers and binding antibody responses. The significance threshold was *p* ≤ 0.05.

## 3. Results

### 3.1. Longitudinal Analysis of Variant-Specific Neutralizing Antibody Responses Revealed Temporal Trends and Stability

**Figure 2:**
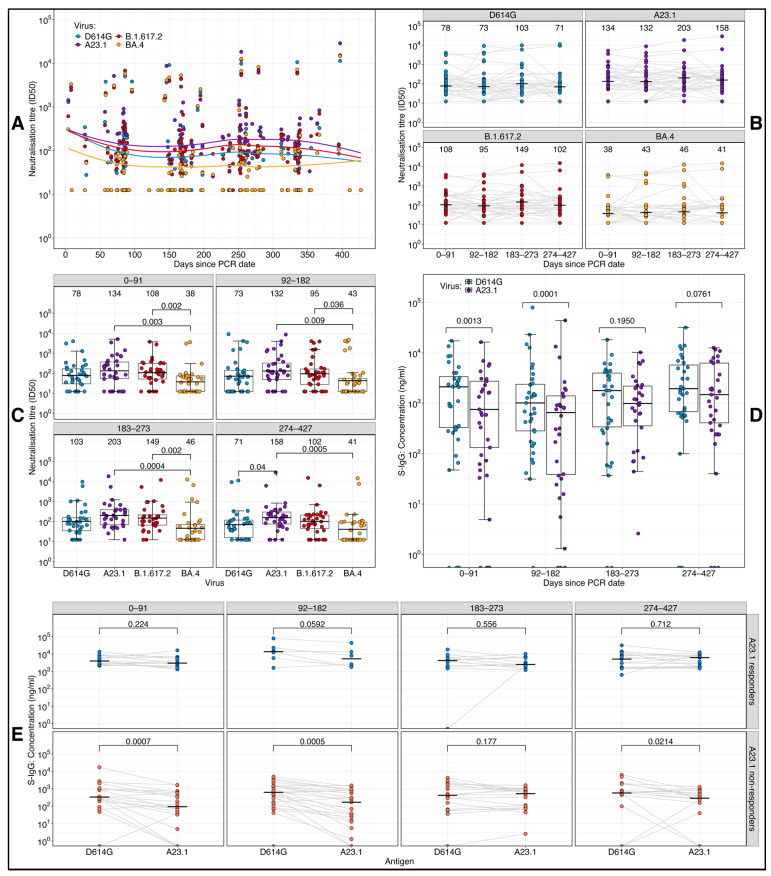
Longitudinal comparison of neutralizing titers and S-IgG levels across SARS-CoV-2 strains. This figure illustrates the longitudinal trends in neutralizing antibody titers and full spike (S)-IgG concentrations across SARS-CoV-2 variants. The line plots show neutralization titers over time for the D614G prototype and the A.23.1, Delta, and BA.4 variants, with the time categorized into four quarterly intervals, 0–91, 92–182, 183–273, and 274–427 days (**A**), and the median titers per quarter (**B**). Boxplots illustrate the distributions of antibody levels displaying medians (horizontal black line) and interquartile ranges (top and bottom of the box) across study groups. Boxplots depict the distribution of neutralizing titers for each virus variant across quarterly time intervals (**C**). Additionally, comparative boxplots illustrate S-IgG concentrations (ng/mL) across the D614G prototype and the A.23.1 variants, analyzed for all study participants (**D**). Using the D614G S-IgG cut-off of 0.432, the data were further stratified by D614G responders and non-responders at each quarterly time point (**E**). Statistical significance was determined at a threshold of *p* ≤ 0.05. Significance bars indicate the *p*-values.

We analyzed neutralizing antibody titers over time against several SARS-CoV-2 variants, A.23.1, D614G, Delta, and BA.4 (Figure 2). Neutralization against BA.4 remained consistently low across the study period (Figure 2A). To provide further insights, we segmented the study into four intervals of approximately 3 months each: 0–91 days, 92–182 days, 183–273 days, and 274–427 days. Boxplot comparisons across these intervals, using an unpaired Wilcoxon test to address missing data (Figure 2B), revealed significantly lower neutralizing titers against BA.4 compared to both A.23.1 and Delta at all time points. At the final time point (274–427 days), A.23.1 titers were significantly higher than those of the D614G variant (*p* < 0.05). The unpaired Wilcoxon test revealed no significant differences in neutralizing titers over time for any of the viral variants, indicating a consistent neutralization pattern throughout the study (Figure 2C). This consistency highlights uniform temporal dynamics of neutralizing antibody responses across intervals, irrespective of the viral variant. However, the boxplot analysis revealed significantly higher S-IgG concentrations against D614G compared to A.23.1 during the early phase of the study (Figure 2D), a trend that persisted even after stratifying the data by D614G responders and non-responders (Figure 2E). These results highlight the variability in neutralizing responses between viral variants and underscore the importance of analyzing immunogenicity in the context of dominant infection waves.

### 3.2. Limited Potency and Breadth of Neutralizing Antibody Responses Against Diverse SARS-CoV-2 Variants, with BA.4 Demonstrating the Highest Immune Evasion

**Figure 3:**
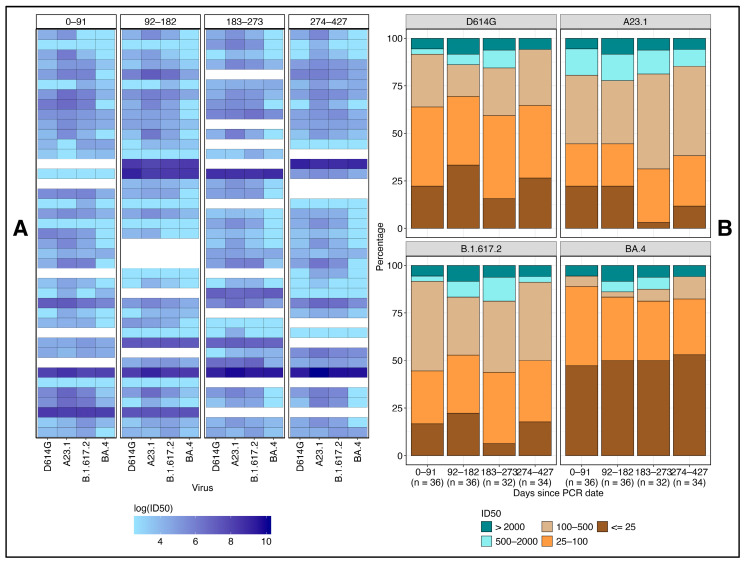
Longitudinal potency and breadth of neutralizing antibody responses against SARS-CoV-2 variants over 427 days. This figure shows a heatmap and bar graph illustrating the neutralizing antibody responses against various SARS-CoV-2 variants over 427 days. In the heatmap (**A**), darker cells represent higher neutralizing titers, while lighter cells indicate lower titers; white cells represent missing data. Each row corresponds to a participant’s neutralization responses over time, highlighting that only a few participants maintained high neutralizing titers across all viruses and time points. The stacked bar graph (**B**) shows the proportion of participants within different neutralization titer groups over time for each variant. Most subjects displayed low (25–100) titers against D614G, intermediate (100–500) titers against A.23.1, and a mix of low and intermediate titers against the Delta variant.

Neutralizing antibody responses against diverse SARS-CoV-2 variants were limited in both potency and breadth over the 427-day observation period. As shown in the heatmap (Figure 3A), only a small subset of participants consistently maintained high neutralization titers across all variants and time points. Notably, BA.4 demonstrated the greatest resistance, with most participants exhibiting low neutralization titers, reflected by lighter heatmap cells. Only a few individuals achieved robust neutralization of this variant, underscoring the challenge of eliciting broad and durable immunity. These findings highlight the ongoing need for vaccines that provide more comprehensive protection against emerging variants.

A stacked-bar graph analysis among participants revealed that neutralizing antibody geometric mean titers (GMTs) were mostly low (25–100) against D614G, while intermediate titers/GMTs (100–500) were more prevalent for A.23.1 across all time points (Figure 3B, Table 2). Delta variant responses primarily fell within the low (25–100) and intermediate (100–500) ranges, whereas BA.4 consistently showed low neutralization titers, highlighting its significant immune evasion compared to the other variants throughout the study period. Notably, only a small fraction of participants achieved high neutralization titers (>2000) across all variants, indicating a generally weak immune response, especially against the highly resistant BA.4 variant.

These findings show the variability in neutralizing antibody potency across SARS-CoV-2 variants, with BA.4 showing the highest resistance to neutralization. The small number of participants exhibiting consistently high titers across all time points highlights the challenge of achieving broad, potent immunity, especially against immune-evasive strains, with implications for vaccine design and long-term immune assessment.

### 3.3. Correlation Between Neutralization Titers and Spike-Specific IgG Binding Responses Across SARS-CoV-2 Variants

**Figure 4:**
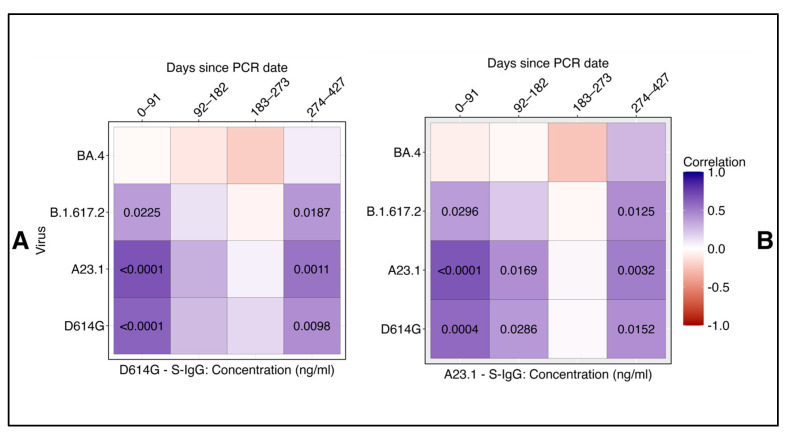
Correlation heat map of neutralizing antibody titers against SARS-CoV-2 variants and spike-directed IgG antibodies. This figure displays heat maps comparing the correlations between neutralizing antibody titers against SARS-CoV-2 variants D614G, A.23.1, Delta, and BA.4 and spike-directed IgG antibody levels for D614G (**A**) and A.23.1 (**B**). Positive correlations are indicated in purple, while negative correlations are shown in red. Statistical significance was determined at a threshold of *p* ≤ 0.05; *p*-values are indicated in the heatmap.

We assessed the correlation between neutralizing antibody titers (ID_50_) and D614G as well as A.23.1 S-IgG concentrations across SARS-CoV-2 variants D614G, A.23.1, Delta, and BA.4 over time using Spearman rank correlation analysis. For the D614G variant, we observed a significant, moderate positive correlation between neutralizing titers (ID50) and S-IgG levels both in the early infection phase (0–91 days) and the later phase (274–427 days) (Figure 4A). Similar trends were noted for A.23.1 and Delta, reinforcing the consistency of the binding–neutralization relationship across these variants. Similarly, for A.23.1 and Delta, consistent correlations were noted at these time points, indicating robust alignment between binding and neutralizing responses. However, a lower correlation was detected during the intermediate phase (92–182 days) for D614G and A.23.1, suggesting a temporary decline in the binding–neutralization relationship (Figure 4B). No significant correlation was found for BA.4 at any time point, highlighting a distinct immune response to this variant.

These findings emphasize the temporal dynamics of antibody binding and neutralizing responses, highlighting the differential correlation between S-IgG levels and neutralization titers across various SARS-CoV-2 variants. The analysis underscores the pivotal role of spike-specific IgG antibodies in mediating protection, particularly against variants like D614G and A.23.1. The distinct lack of correlation observed for BA.4 further illustrates its unique immunogenic properties, suggesting potential immune evasion. This divergence has significant implications for vaccine development and future therapeutic strategies, particularly in addressing variants capable of escaping immune recognition.

### 3.4. Comparative Longitudinal Analysis of GMT Fold Changes and Neutralization Titers in Reinfected and Vaccinated Subjects

**Figure 5:**
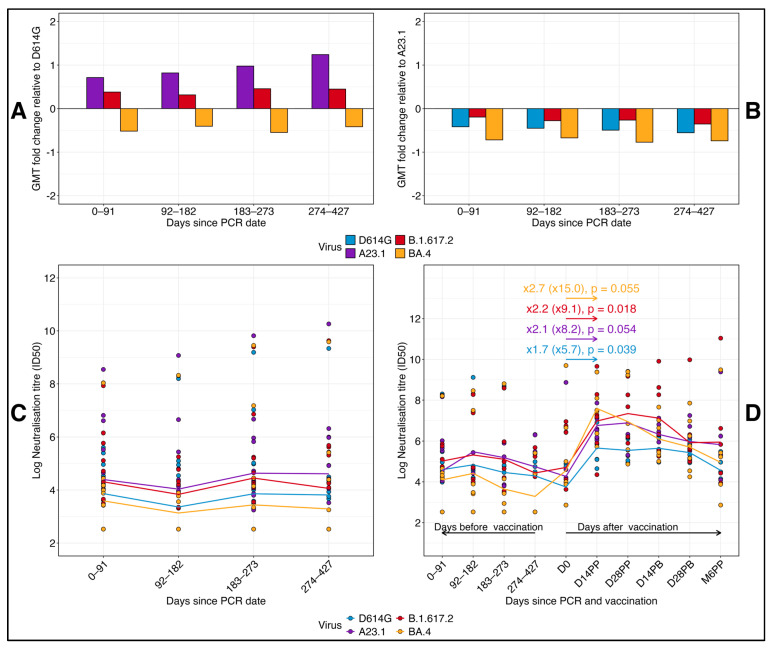
Fold changes in neutralizing titers relative to D614G and A.23.1 and neutralization patterns in re-infected and vaccinated Subjects. This figure shows the log-transformed geometric mean titer (GMT) fold changes in neutralizing antibody responses relative to D614G (**A**) and A.23.1 (**B**) across different time points in a cohort of 41 participants. A fold change above 0 indicates an increase, while a fold change below 0 indicates a decrease in neutralizing titers relative to the reference virus. Panel (**C**) displays the neutralizing titers of re-infected subjects over time, categorized by days post-infection, with a focus on variations in neutralization responses across different virus variants. (**D**) illustrates the neutralizing titers before and after vaccination in vaccinated subjects, showing the comparative changes in responses to each virus variant following vaccination. Horizontal lines represent the log-transformed geometric mean titers at each time point for each virus. Fold changes are presented as log-transformed values, with raw unlogged values in brackets. Statistically significant differences are indicated, marked by *p*-values ≤ 0.05, confirming a consistent rise in antibody titers across all variants following vaccination.

We performed a comparative analysis of geometric mean titer (GMT) fold changes relative to D614G and A.23.1 across all time points, as well as neutralizing titers for 13 reinfected subjects and pre- and post-vaccination titers in 8 ChAdOx1-S-vaccinated participants (Figure 5). At each time point, fold changes in neutralizing titers were calculated for each virus variant relative to D614G (Figure 5A) and A.23.1 (Figure 5B). A fold change greater than 0 indicated an increase in neutralizing titers, while a fold change below 0 represented a decrease. A fold change of 0 implied no change in titers. When neutralizing titers were compared to D614G, Delta exhibited a marginal increase across all time points, indicating a slight enhancement in the neutralizing response. Over time, A.23.1 demonstrated a gradual fold increase in neutralizing titers compared to D614G, signifying consistently higher titers for A.23.1. In contrast, BA.4 showed a consistent fold decrease relative to D614G, indicating lower neutralizing antibody responses for BA.4 at all time points.

Relative to A.23.1 (Figure 5B), all other virus variants showed a fold decrease across all time points, highlighting the superior neutralizing response elicited by A.23.1. This finding suggests that A.23.1 maintained a stronger neutralizing response compared to D614G, Delta, and BA.4 throughout the study period. Neutralizing titers for reinfected subjects, defined by an 11-fold or higher increase in N-IgG concentrations after the initial peak, were assessed (Figure 5C). A total of thirteen subjects met the reinfection criteria. For these individuals, neutralizing titers displayed a declining trend during the first 182 days post-primary infection, followed by a gradual increase between 183 and 273 days across all virus variants. In a separate analysis of eight participants who received both doses of the AZN vaccine, we compared neutralizing titers before and after vaccination (Figure 5D). BA.4 titers, which were initially low post primary infection, rose sharply by 15-fold after vaccination. Delta titers increased by nine-fold, D614G by six-fold, and A.23.1 by two-fold. These results highlight the strong boosting effect of vaccination, enhancing neutralizing responses against all variants, including BA.4, which had exhibited persistently lower responses pre-vaccination.

These findings highlight the dynamic nature of neutralizing antibody responses across SARS-CoV-2 variants, revealing significant variability in immune recognition. The analysis underscores the profound enhancement in immune responses following vaccination, particularly against variants with initially low neutralization profiles. The substantial post-vaccination increases in neutralizing titers against BA.4, Delta, and D614G demonstrate the critical role of vaccination in bolstering the breadth of neutralizing antibody responses. Furthermore, the consistently higher neutralizing titers observed against A.23.1, both before and after vaccination, reinforce the observed effect of immune imprinting from the primary A.23.1 SARS-CoV-2 infection in this population. These findings have important implications for understanding variant-specific immune responses and guiding future vaccination strategies.

## 4. Discussion

This study aimed to investigate the long-term dynamics of neutralizing antibody responses in a Ugandan cohort, with a specific focus on the influence of early exposure to the A.23.1 SARS-CoV-2 variant on subsequent immune responses to both natural infections and vaccinations with the wild-type prototype-based vaccines. Given the unique exposure history of this cohort, which first encountered the A.23.1 variant before the more globally dominant Delta and Omicron (BA.4) variants emerged, this study was justified by the need to explore the effects of immune imprinting in an African population and its broader implications for public health and vaccine design strategies. We hypothesized that initial infection with A.23.1 would leave an immunological imprint that could influence the magnitude, specificity, and durability of neutralizing responses to subsequent infections and vaccinations. Our primary finding was the sustained and robust neutralizing antibody response against the A.23.1 variant, which surpassed responses to D614G, Delta, and BA.4. This highlights the potential impact of initial antigenic exposure on shaping long-term immune responses and is supported by previous observations of preferential neutralization of the primary infecting variant over other variants [23]. Additionally, neutralizing responses to BA.4 were significantly weaker across all time points, underscoring the unique immune-evasive and antigenic divergence properties of this variant, consistent with findings from global studies on Omicron variants [24,25]. The higher neutralizing titers against A.23.1 throughout indicate a lasting imprint that may confer enhanced protection against this variant. These results align with the concept of immune imprinting, where the first antigen encounter primes the immune system in ways that influence responses to future exposures [26].

This study primarily investigates the A.23.1 variant, first identified in Uganda, and its impact on immune responses to subsequent variants, including Delta and Omicron (BA.4). While other early variants of concern (VOCs), such as Beta (B.1.351) and Gamma (P.1), possess the E484K mutation in the RBD and confer stronger immune evasion compared to Alpha (N501Y) [27], these variants did not play a significant roles in Uganda’s early SARS-CoV-2 epidemic waves. Instead, lineage A.23 dominated the initial infections, evolving into A.23.1 before transitioning to Delta and, later, Omicron. Genomic surveillance data show that Alpha, Beta, and Gamma VOCs circulated at minimal circulation in Uganda [21], thus limiting the opportunity to assess immune responses to these variants in our cohort. Despite this, immune imprinting effects observed with A.23.1 are consistent with findings from other populations exposed to different primary variants. For example, research in South Africa showed that initial exposure to Beta influenced subsequent immune responses differently from those who first encountered the ancestral Wuhan-1 strain [28]. Similarly, studies in the UK and China demonstrated that early infection with specific SARS-CoV-2 variants shaped preferential neutralization patterns against related strains [29,30], a phenomenon also confirmed in controlled animal models [31]. These findings underscore the broader relevance of immune imprinting across diverse epidemiological settings, emphasizing the need for tailored vaccine strategies that consider early viral exposures. Understanding these dynamics is crucial for optimizing future vaccine formulations and ensuring robust protection against emerging variants.

Our findings revealed that despite prior imprinting by the A.23.1 variant, vaccination in previously infected individuals substantially increased neutralizing antibody titers and breadth across all tested variants. However, neutralizing antibody responses to A.23.1 remained higher than those against D614G, further highlighting the A.23.1 imprint. This suggests that prior exposure to A.23.1 did not diminish the potency and breadth of the vaccination response, even in the presence of a response favoring the primary infection variant over the variant of the vaccine antigen, thus highlighting the effectiveness of vaccines in enhancing and broadening immunity. This aligns with other studies showing that COVID-19 vaccines can elicit broad and potent neutralizing responses regardless of prior variant exposure [12]. Notably, our data also revealed a pronounced reduction in neutralizing capacity against the BA.4 variant at all time points, aligning with this variant’s potential for immune evasion [32]. These results corroborate prior studies demonstrating diminished vaccine efficacy and reduced antibody neutralization against Omicron subvariants [33,34,35], underscoring the necessity for ongoing surveillance and potential vaccine updates to counter evolving viral threats. Our findings introduce a crucial dimension to the literature on immune imprinting and SARS-CoV-2. While previous studies focused on the priming effect of the original Wuhan-1 strain and subsequent variants of concern, the impact of early circulating variants, such as A.23.1, was overlooked. This study bridges the gap by examining the A.23.1 variant, revealing how other non-Wuhan-1 strains like A.23.1 influence long-term immune responses.

A key strength of this study was its longitudinal design, which allowed for extended assessment of immune responses, offering a dynamic view of antibody kinetics. The combined use of binding and neutralizing antibody assays, alongside multiple SARS-CoV-2 variants, enhanced the robustness and comprehensiveness of our findings. However, the relatively small sample size and the predominance of males and asymptomatic participants could introduce bias and limit generalizability. Nonetheless, these demographics accurately represent those most affected by COVID-19 in sub-Saharan Africa.

The differential neutralizing antibody responses observed against various SARS-CoV-2 variants suggest that the initial exposure to A.23.1 may have induced a unique immunological imprint, potentially through mechanisms like epitope specificity and B-cell memory formation [35]. The lack of a significant correlation between S-IgG concentrations and neutralizing titers for the BA.4 variant further highlights the evolving challenges posed by new SARS-CoV-2 strains [36]. Clinically, our results suggest that individuals initially exposed to the A.23.1 variant may have enhanced protection against similar variants but could be less protected against highly divergent strains like BA.4. This supports the need for variant-specific boosters to ensure broad and effective immunity. Moreover, the demonstrated boost in neutralizing antibodies following vaccination in previously infected individuals supports the continued use of vaccines to enhance and diversify immune responses in populations with prior SARS-CoV-2 exposure. The diminished sensitivity of the BA.4 Omicron variant was anticipated, as Omicron variants possess up to 15 mutations in the receptor-binding domain (RBD) [37,38]. Mutations in Omicron, such as K417N, G446S, E484A, and Q493R, have been shown to significantly compromise the neutralizing ability of RBD-binding antibodies whose epitopes overlap with the ACE2-binding motif.

## 5. Study Limitations

While this study provides valuable insights into the long-term immune consequences of initial SARS-CoV-2 A.23.1 exposure, several limitations should be acknowledged. First, the sample size of 41 participants, although carefully characterized and followed longitudinally, is relatively small, which may limit the generalizability of our findings. However, the depth of our immunological assessments across an extended period strengthens the interpretation of immune imprinting effects. While the sample size is modest, our approach ensures robust statistical comparisons by leveraging repeated measurements per participant over time, thus enhancing the statistical power of our analysis. To validate our findings, we applied stringent statistical corrections, including the Wilcoxon test with Bonferroni adjustments for multiple comparisons and Spearman correlation analyses to assess binding and neutralization relationships. Second, the cohort was predominantly comprised of young, male truck drivers who were asymptomatic or mildly symptomatic. This demographic representation, although reflective of Uganda’s early outbreak dynamics, may not fully capture immune responses in older or more clinically severe cases. Future studies should expand to more diverse cohorts to validate these findings across broader populations. Third, while we analyzed neutralizing antibody responses against key SARS-CoV-2 variants (A.23.1, D614G, Delta, and BA.4), earlier VOCs, such as Alpha, Beta, and Gamma, were not included due to their low prevalence in Uganda at the time of study recruitment, potentially limiting direct comparisons with global immune imprinting patterns. Finally, while neutralizing antibodies are a critical correlate of protection, additional functional immune parameters, such as T-cell responses, were not assessed in this study. Future work incorporating cellular immunity will be essential to provide a more comprehensive understanding of immune imprinting and long-term immunity.

## 6. Conclusions

In conclusion, this study provides critical insights into the long-term immune consequences of primary SARS-CoV-2 A.23.1 exposure, demonstrating that early viral encounters shape the breadth, magnitude, and durability of neutralizing antibody responses to subsequent infections and vaccinations. Our findings highlight the persistence of strong neutralizing responses against A.23.1, contrasted by significantly reduced cross-neutralization against immune-evasive variants, such as Omicron BA.4. While vaccination effectively boosted immunity across all tested variants, the imprint of A.23.1 remained evident, reinforcing the role of early antigenic exposure in shaping long-term immune trajectories. These results underscore the necessity of continuous antigenic surveillance and periodic vaccine updates to address evolving variants with enhanced immune escape potential. Furthermore, this study emphasizes the importance of tailored immunization strategies that consider population-specific exposure histories to optimize vaccine effectiveness. Future research integrating cellular immunity and expanding cohort diversity will be crucial to fully unravel the complexities of immune imprinting and its implications for global SARS-CoV-2 vaccine and public health strategies.

## Figures and Tables

**Table 1:** Characteristics of study participants, *n* = 41.

Characteristics	*n* (%)
Total participants	41 (100)
Age, years (Median, IQR)	29.0 (23.0–34.0)
Gender	
Female	9 (22.0)
Male	32 (78.0)
Symptoms	
Yes *	4 (9.8)
No	25 (61.0)
Unknown	12 (29.3)

* Mild symptoms were chest pain and shortness of breath (*n* = 1), cough and runny nose (*n* = 1), fever (*n* = 1), and headache (*n* = 1).

**Table 2:** Proportion of subjects neutralizing SARS-CoV-2 variants at various neutralizing titers over time.

Time Point (Days)		ID50 ≤ 25 Limited	25–100 Low	100–500 Intermediate	500–2000 High	ID50 > 2000 Robust
0–91	D614G	8 (22.2)	15 (41.7)	10 (27.8)	1 (2.8)	2 (5.6)
	A.23.1	8 (22.2)	8 (22.2)	13 (36.1)	5 (13.9)	2 (5.6)
	Delta	6 (16.7)	10 (27.8)	17 (47.2)	1 (2.8)	2 (5.6)
	BA.4	17 (47.2)	15 (41.7)	2 (5.6)	0 (0)	2 (5.6)
92–182	D614G	12 (33.3)	13 (36.1)	6 (16.7)	2 (5.6)	3 (8.3)
	A.23.1	8 (22.2)	8 (22.2)	12 (33.3)	5 (13.9)	3 (8.3)
	Delta	8 (22.2)	11 (30.6)	11 (30.6)	3 (8.3)	3 (8.3)
	BA.4	18 (50.0)	12 (33.3)	1 (2.8)	2 (5.6)	3 (8.3)
183–273	D614G	5 (15.6)	14 (43.8)	8 (25.0)	3 (9.4)	2 (6.3)
	A.23.1	1 (3.1)	9 (28.1)	16 (50.0)	4 (12.5)	2 (6.3)
	Delta	2 (6.3)	12 (37.5)	12 (37.5)	4 (12.5)	2 (6.3)
	BA.4	16 (50.0)	10 (31.3)	2 (6.3)	2 (6.3)	2 (6.3)
274–427	D614G	9 (26.5)	13 (38.2)	10 (29.4)	0 (0)	2 (5.9)
	A.23.1	4 (11.8)	9 (26.5)	16 (47.1)	3 (8.8)	2 (5.9)
	Delta	6 (17.6)	11 (32.4)	14 (41.2)	1 (2.9)	2 (5.9)
	BA.4	19 (55.9)	9 (26.5)	4 (11.8)	0 (0)	2 (5.9)

This table presents the number and corresponding proportions of subjects exhibiting varying levels of neutralizing titers against each SARS-CoV-2 variant over time. The table categorizes subjects into distinct neutralization levels, illustrating their distribution across distinct time intervals: 0–91 days, 92–182 days, 183–273 days, and 274–427 days. This breakdown provides an overview of the participants’ responses at low, intermediate, and high neutralization titers for each virus variant, enabling the comparison of neutralization patterns throughout the study period.

## Data Availability

The raw data supporting the conclusions of this article will be made available by the authors upon request from the corresponding author (JSer).
